# Integration of Light and Brassinosteroid Signaling during Seedling Establishment

**DOI:** 10.3390/ijms222312971

**Published:** 2021-11-30

**Authors:** Fang Lin, Jing Cao, Jiale Yuan, Yuxia Liang, Jia Li

**Affiliations:** Ministry of Education Key Laboratory of Cell Activities and Stress Adaptations, School of Life Sciences, Lanzhou University, Lanzhou 730000, China; caojing94@163.com (J.C.); jialeyhh@163.com (J.Y.); liangyx19@lzu.edu.cn (Y.L.); lijia@lzu.edu.cn (J.L.)

**Keywords:** photomorphogenesis, skotomorphogenesis, light, brassinosteroids, brassinolide, Arabidopsis

## Abstract

Light and brassinosteroid (BR) are external stimuli and internal cue respectively, that both play critical roles in a wide range of developmental and physiological process. Seedlings grown in the light exhibit photomorphogenesis, while BR promotes seedling etiolation. Light and BR oppositely control the development switch from shotomorphogenesis in the dark to photomorphogenesis in the light. Recent progress report that substantial components have been identified as hubs to integrate light and BR signals. Photomorphogenic repressors including COP1, PIFs, and AGB1 have been reported to elevate BR response, while photomorphogenesis-promoting factors such as HY5, BZS1, and NF-YCs have been proven to repress BR signal. In addition, BR components also modulate light signal. Here, we review the current research on signaling network associated with light and brassinosteroids, with a focus on the integration of light and BR signals enabling plants to thrive in the changeable environment.

## 1. Introduction

As sessile and photoautotrophic organisms, higher plants have evolved a sophisticated system to adjust their growth and development in response to the internal signals and external cues [1]. Sailing the sea depends on the helmsman, while all living things grow under the sun. Among the environment stimuli, sunlight is a fundamental necessity for plant photosynthesis and an essential signal for developmental programs, including seed germination, seedling photomorphogenesis, phototropism and gravitropism, shade-avoidance, floral transition, and circadian rhythm. Upon seed germination, plants exhibit short hypocotyls, opened and green cotyledons in the light, which is known as photomorphogenesis or de-etiolation. Whereas seedlings develop long hypocotyls, apical hooks, and closed and etiolated cotyledons in darkness are called skotomorphogenesis or etiolation [1,2,3].

Brassinosteroids (BRs) are a group of steroidal phytohormones crucial for a broad range of developmental and physiological processes including seed germination, seedling morphogenesis, stomata development, vascular tissue differentiation, male fertility, flowering, and senescence [4,5]. In the presence of BR, seedlings display long hypocotyls, closed cotyledons, and decreased chlorophyll. However, BR-deficient or insensitive mutants in the dark cause typical photomorphogenesis with shortened hypocotyls, opened and expanded cotyledons [6]. Light and BRs play antagonistic role in seedling morphogenesis. The two morphological changes are the major typical results of light information and/or BRs responses, which have been an excellent model system for studying the interconnection between light and brassinosteroids. Extensive studies have revealed the complicated but delicate light and BR pathways in regulating plant growth and development [7,8,9,10,11]. A number of significant studies have been conducted to elucidate the close link between light and BR cascades in recent years [12,13,14,15,16,17,18,19]. Here, we summarize the light and BR signal events in seedling morphogenesis, as well as provide an overview of the molecular framework for oppositely regulation of seedling morphogenesis by light and BRs. For details on the integration of light and brassinosteroids in the regulation of biotic and abiotic stress and other developmental processes, readers are directed to excellent recent reviews [20,21,22,23].

## 2. Regulation of Seedlings Morphogenesis by Light Information

**Figure 1:**
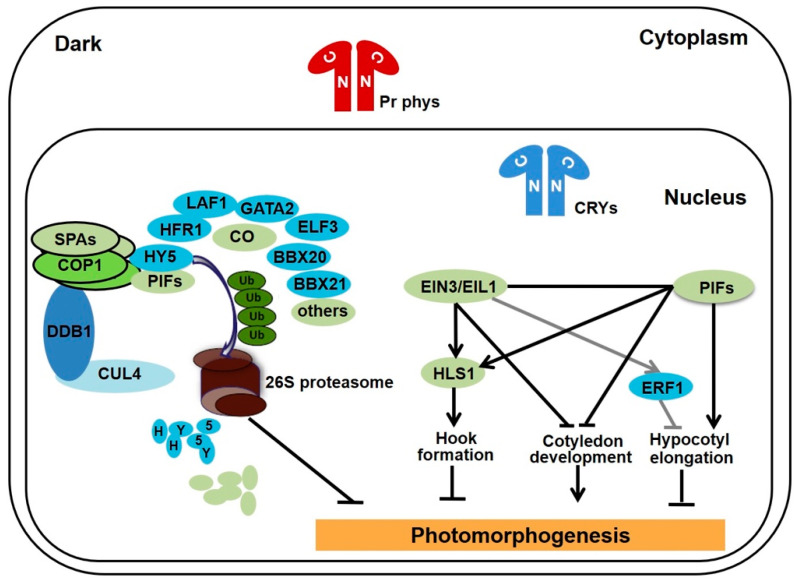
Photomorphogenic repressors promote skotomorphogenesis in the dark.

Plants employ a series of photoreceptors to precisely perceive light and modulate their growth and development [3,24,25]. Phytochromes sense red/far-red light (600–750 nm), which possess five members PHYTOCHROME A-E (phyA-E) in Arabidopsis. The UV-A/blue light (350–500 nm) receptors are CRYPTOCHROMES (CRYs), phototropins, and the ZEITLUPE/FLAVIN-BINDING KELCH REPEAT F-BOX 1/LOV KELCH PROTEIN 2 (ZTL/FKF1/LKP2) family members. The UV-B light (275–320 nm) is perceived by UV RESISTANCE LOCUS 8 (UVR8) sensor. When photoreceptors are inactive in the dark, photomorphogenic repressor CONSTITUTIVELY PHOTOMORPHOGENIC 1 (COP1)-SUPPRESSOR OF PHYTOCHROME A (SPA) complex promote skotomorphogenesis by targeting photomorphogenesis-promoting transcription factors such as ELONGATED HYPOCOTYL 5 (HY5), LONG HYPOCOTYL IN FAR-RED 1 (HFR1) and B-BOX PROTEIN 21 (BBX21) for ubiquitination and degradation [26,27,28]. Another group of photomorphogenic repressors are PHYTOCHROME-INTERACTING FACTORS (PIFs), a class of bHLH-type transcription factors, that inhibit photomorphogenesis by directly regulating the expression of myriad of downstream target genes [29]. Furthermore, ETHLENE-INSENSITIVE 3 (EIN3)/EIN3-LIKE 1 (EIL1), master transcription factors that initiate transcriptional cascades for ethylene response, have been proved to be the third kind of photomorphogenic repressors, especially in the process of hook formation and cotyledon closed in darkness (Figure 1) [30].

In the dark, red light-absorbing phytochromes retain in the cytoplasm, while inactive cryptochromes present in the nucleus. The complex of CUL4-DDB1-COP1-SPAs E3 ligase target photomorphogenesis-promoting transcription factors, such as HY5, HFR1, and BBX21, for ubiquitination and degradation via the 26S proteasome. Meanwhile, PIFs and EIN3/EIL1 cooperatively regulate hypocotyl elongation and confine cotyledon open and expand, as well as induce hook formation via promoting the expression of *HLS1*. Thus, COP1-SPA, PIFs, and EIN3/EIL1 are the master photomorphogenic repressors to maintain the etiolated phenotypes of seedlings in darkness.

Upon visible light absorption, active photoreceptors negatively regulate the photomorphogenic-repressors to facilitate photomorphogenesis. COP1 is one of the most extensively characterized photomorphogenic repressors, which harbors an N-terminal RING-finger motif, a medial coiled-coil domain, and seven WD40 repeats at its C-terminus [31]. SPA proteins carry Ser/Thr kinase domain, coiled-coil domain, and WD40-repeat domain [32]. COP1 is a single-copy gene, while there are four SPA members (SPA1-4) with partially redundant functions. Both *cop1* mutants and *spa* quadruple (*spaQ*) mutants exhibit constitutively photomorphogenic phenotype in the dark, mimicking the light-grown wild type seedlings [32,33]. In Arabidopsis, two COP1 molecules and two SPA molecules form a tetrameric complex via their respective coiled-coil domains [34]. With the help of RING BOX PROTEIN 1 (RBX1)-CULLIN4 (CUL4)-DNA DAMAGE-BINDING PROTEIN 1 (DDB1), COP1 functions as a high order RING type E3 ubiquitin complex CUL4-DDB1-COP1-SPA, which accelerate a myriad of substrates for ubiquitination and degradation by 26S proteasome (Figure 1) [35,36]. Additionally, COP9 signalosome (CSN) complex which is composed of eight subunits (CSN1-8) and CDD complex consisting of COP10, DDB1, and DE-ETIOLATED 1 (DET1) have been identified to repress photomorphogenesis in the dark [37]. Loss-of-function of *CSN* and *CDD* complex develop constitutively photomorphogenic phenotype in darkness. Therefore, CSN, COP1, COP10, and DET1 are members of COP/DET/FUS proteins which function as photomorphogenic repressors. CSN controls the RUB/NEDD8 modification of Cul4-based E3 ligases such as Cul4-DDB1-CDD and Cul4-DDB1-COP1-SPA complex to repress photomorphogenesis [38].

**Figure 2:**
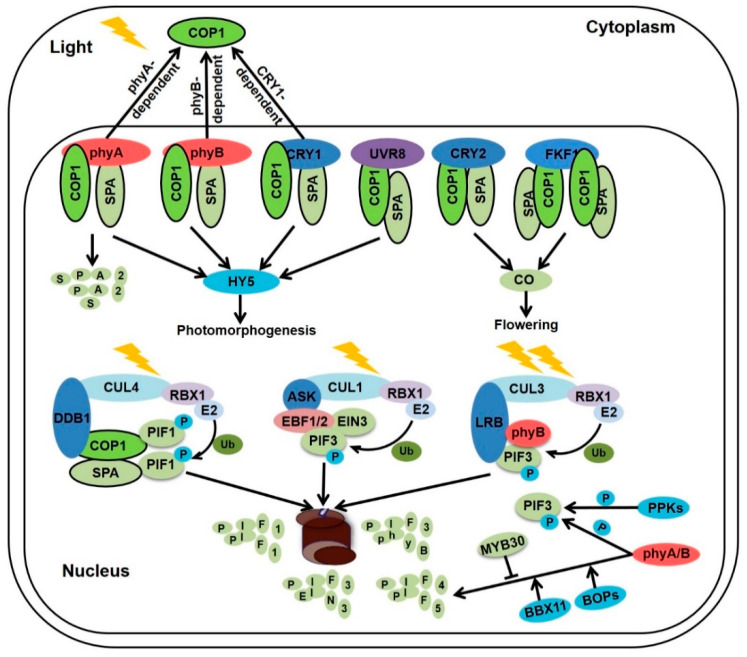
Light induces the inactivation or degradation of photomorphogenic repressors.

In the red/far-red light response, biologically active far-red light-absorbing (Pfr) form of phytochrome A and phytochrome B directly associate with SPA1 to disrupt the physical interaction between COP1 and SPAs, resulting in reorganizing the COP1-SPAs complex and repressing its ability to ubiquitinate and degrade photomorphogenesis-promoting transcription factors (Figure 2) [39,40]. Blue light-activated CRY1 interacts with SPA1 to facilitate the dissociation of COP1-SPA1 complex. However, CRY2 associates with SPA1 to promote the CRY2-COP1 interplay [41,42,43]. Recently, it is proved that the VP motif of CRY2 interacts with COP1 to compete with COP1 substrates and repress COP1 ubiquitin ligase activity for its targets [44]. Similar with CRY2, UV-B activated UVR8 monomer exerts its VP motif to interact with COP1, which competitively inhibits the binding between COP1 and its substrates [45]. Meanwhile, traditional CUL4-based COP1-SPA E3 apparatus disassociated to UVR8-COP1-SPA complex and CUL4-DDB1-RUB1/2 E3 complex [46,47]. The newly established CUL4-DDB1-RUB1/2 E3 complex promotes HY5 degradation, while COP1 interacts with RUB1/2 and facilitates the ubiquitination and degradation of RUP1/2 to balance the accumulation of RUP1/2 in the prolonged UV-B-induced photomorphogenesis [46]. Therefore, UVR8 monomer associates with COP1 to elevate HY5 accumulation through blocking the interaction between COP1 and HY5, as well as targeting RUB1/2 for degradation in the UV-B response. Moreover, FKF1 is reported to interact with the RING domain of COP1 to inhibit the COP1 dimerization, ultimately inactivating the COP1-SPA complex dependent CO degradation to regulate flowering process and partially inhibiting COP1-mediated hypocotyl elongation [48]. In addition, COP1 translocates from the nucleus to the cytoplasm, declining its nuclear abundance in the constant visible light conditions. It has been documented that the half-life of nucleocytoplasmic partition is 2.4 ± 0.5 h [2,49]. Furthermore, phyA-dependent COP1 ubiquitinates SPA2 for degradation, which also contributes to light-triggered COP1-SPA repression [50]. Taken together, upon exposure to light, activated photoreceptors promote the disassociation of COP1-SPA complex and COP1 dimerization, degradation of SPA2, COP1 translocation from the nucleus to the cytoplasm, and competitively binding with COP1 substrates through the VP motif of photoreceptors [2,39,40,41,42,43,44,45,51], reducing the ubiquitin ligase activity of COP1 in the nucleus and allowing photomorphogenesis (Figure 2).

Another group of photomorphogenic repressors called PIFs, among which, PIF1/3/4/5/7 and PIF8 contain an active phytochrome B (APB) motif which are capable of interacting with phyB, while PIF1 and PIF3 also have an active phytochrome A (APA) motif that can bind phyA. Many reports show that phytochromes and cryptochromes promote the inactivation or degradation of PIFs in certain light conditions to facilitate photomorphogenesis [52,53,54,55]. Photo-activated phyB translocates from the cytoplasm to the nucleus and directly interacts with PIF3/4/5, which promotes the following phosphorylation and degradation [54,56,57,58,59]. Phytochromes and PHOTOREGULATORY PROTEIN KINASEs (PPKs) contribute to the light-induced PIF3 phosphorylation. EIN3-BINDING F-BOX PROTEIN 1/2 (EBF1/2) target PIF3 for ubiquitination and degradation in the wide range of light intensity, whereas LIGHT-RESPONSE BRIC-A BRACK/TRAMTRACK/BROADs (LRBs) promote PIF3 and phyB degradation under high-light condition [54,58]. Both PIF1 and PIF8 repress photomorphogenesis and seed germination [60,61]. PIF1 directly interacts with COP1-SPA complex to repress photomorphogenesis in a synergistic manner [27,62]. The active Pfr form of phyB interacts with PIF1 and recruits SPA1 to phosphorylate PIF1, subsequently CUL4 based COP1-SPA E3 ubiquitin ligase capture PIF1 for ubiquitylation and degradation in the red light [60,63]. PIF8 associates with phyB which promotes the degradation of PIF8 in the red light, whereas PIF8 accumulates to high level in the far-red light which dependents on the phyA-mediated COP1 inhibition, ultimately repressing phyA-induced seed germination and photomorphogenesis [61].

PIF4 and PIF5 not only repress photomorphogenesis, but also promote thermomorphogenesis and shade avoidance [59,64]. PIF4 directly interacts with the pfr form of phyB via the APB motif. Although lacking the APA motif, PIF4 has weak affinity with phyA. The stability of PIF4 is regulated by phyB. Upon red light irradiation, phyB translocates from the cytoplasm to the nucleus to bind PIF4, which subsequently promotes its phosphorylation and degradation [59]. BLADE-ON-PETIOLE (BOP) proteins have been reported to act as substrate adaptors in a CUL3 E3 ligase complex to mediate the degradation of PIF4 [65]. Similar to PIF4, red light also induces the rapid phosphorylation and degradation of PIF5 via the ubiquitin-proteasome system. Both phyA and phyB redundantly dominate this process, though PIF5 can only interact with light-activated phyB [59,66]. However, PIF4 and PIF5 reaccumulation under prolonged R light irradiation which dependent on MYB30 in response to dynamic light environment [67]. BBX11 has been reported to physically interacts with phyB and PIF4. BBX11 not only enhances the interaction of phyB with PIF4 to repress the reaccumulation of PIF4 in the red light, but also attenuates the transcriptional activity of PIF4 to repress its target gene expression. Thus, BBX11 negatively regulates PIF4 protein accumulation and biochemical activity to promote photomorphogenesis [68]. BRASSINOSTEROID INSENSITIVE 2 (BIN2) is a glycogen synthase kinase-3 (GSK3)-like kinase which has been found to phosphorylate PIF4 independent of light. Besides TOPP4, a type one protein phosphatase, dephosphorylates PIF5 to hinder the ubiquitin-mediated degradation of PIF5 [69]. In addition, FyPP1 and FyPP3, two subunits of PROTEIN PHOSPHATASE 6 (PP6), may function as the phosphatases that dephosphorylate PIF3/4 and alleviate the PIF4 degradation [11,70]. However, the kinase and E3 ligase that mediate red light-induced PIF4 and PIF5 phosphorylation and degradation are yet to be identified. In the blue light, CRY1 physically interacts with PIF4 to confine the transcriptional activity of PIF4 which arrests high temperature-mediated hypocotyl elongation [53,55]. However, high temperature not only disassociates TCP DOMAIN PROTEIN 17 (TCP17) from CRY1 to release the transcriptional activity of TCP17, but also increases the stability of TCP17 and TCP5. The accumulated and activated TCP17 and TCP5 up-regulate the expression of *PIF4*, as well as interact with PIF4 to reinforce the transcriptional activity of PIF4, leading to the thermomorphogenesis [71,72]. Furthermore, TCP17 along with TCP5 and TCP13 play essential role in shade-triggered auxin biosynthesis through elevating the transcriptional level of *PIF4/5* and *YUC2/5/8* [73]. Recently, PIF4 is found to bind to the G-box motif of its own promoter to activate *PIF4* transcription, whereas CRY1 binds PIF4 to inhibit the self-activated transcription of PIF4 to suppress shoot branching [74]. Under low blue light, CRY1 and CRY2 contact with PIF4 and PIF5 to modulate their transcriptional activity to facilitate growth through binding to the common DNA element shared with PIF4/5 [55]. The destabilized PIF1/3/4/5 and weak-activated PIF4/5 attenuate skotomorphogenic-promoted gene expression and thus contribute to de-etiolation.

Red/far-red light-activated phytochromes and blue light-excited CRY1 interact with SPA1 to disassociate COP1-SPAs complex, while blue light-excited CRY2 associates with SPA1 to enhance the interaction between COP1 and CRY2 which is a competitor of COP1 substrates. Light-activated FKF1 interacts with COP1 to inhibit COP1 dimerization. In the UV-B irradiation, UVR8 monomer which carries a VP motif competitively interacts with COP1 to release the traditional COP1 substrates. Besides, the phyA and COP1 dependent SPA2 degradation contributes to a decrease in the COP1-SPAs E3 ligase activity in the light. In addition, phytochromes and cryptochrome promote COP1 translocation from nucleus to the cytosol in continues light condition. CUL4-DDB1-COP1-SPAs complex promotes PIF1 phosphorylation and degradation. At the beginning of light exposure, plants sense weak light to promote PIF3 phosphorylation via phytochromes and PPKs. Phosphorylated PIF3 recruits EBF1/2 and CUL1 to form SCF^EBF1/2^-PIF3 complexes, leading to the ubiquitination and degradation of PIF3. Under strong light treatment, CUL3^LRBs^ E3 ligase also triggers PIF3 degradation in addition to SCF^EBF1/2^. It is worth to note that the CUL3^LRBs^ E3 ligases results in the degradation of both phyB and PIF3 to attenuate and balance plant light responses. Moreover, phytochrome A and phytochrome B promote PIF4 and PIF5 phosphorylation and degradation. MYB30 negatively regulates the reaccumulation of PIF4 and PIF5 under prolonged red light irradiation, whereas BBX11 positively controls PIF4 reaccumulation in the prolonged red light.

PIF7 is considered as a master regulator of shade-induced hypocotyl elongation [75,76]. When grown in dense stands, seedlings elongate their hypocotyl in an effort to reach light which is a process termed shade avoidance. A reduction in the ratio of red to far-red light and low blue light invoke shade avoidance response which is perceived by phytochromes and cryptochromes. PIF7 plays an essential role in low red:far-red light mediated shade avoidance. In contrast to PIF1/3/4/5, PIF7 is relatively light-stable. Far-red light absorbing form of phyB interacts with phosphorylated PIF7. In the meantime, 14-3-3 proteins bind and retain phosphorylated PIF7 in the cytoplasm, resulting in the repression of PIF7 target gene expression and inhibition of hypocotyl elongation. However, shade-induced the decrease of active form of phyB and accumulation of de-phosphorylated PIF7 in the nucleus, leading to shade-triggered hypocotyl elongation [75]. Thereby, the nuclear import of de-phosphorylated PIF7 is triggered by shade response, which is suppressed by 14-3-3 proteins-mediated retention of PIF7 in the cytoplasm [77]. More detailed work on the kinase and phosphatase of PIF7 will help us to better understand the PIF7-mediated shade-triggered hypocotyl elongation.

The third group of photomorphogenic repressor known as EIN3/EIL1, which are subjected to the ubiquitination and degradation executed by SCF^EBF1/2^ E3 ligase complex (Figure 2) [78]. Etiolated seedlings employ EIN3/EIL1 to promote hook formation and cotyledon closing; however, EIN3 activates *ERF1* gene expression to inhibit hypocotyl elongation in the presence of ethylene in darkness. In addition, EIN3 directly binds to the promoter of *PIF3* and activates its gene expression to mediate the ethylene-induced hypocotyl elongation in the light [79]. Thus, EIN3/EIL1 act as photomorphogenic repressors in the process of hypocotyl elongation in the light, hook formation and cotyledon closing in darkness, while EIN3/EIL1 function as photomorphogenesis-promoting factors to inhibit hypocotyl elongation in darkness. When grown under the soil, darkness and mechanical pressure stimulate seedlings to fulfil the skotomorphogenic developmental program for survival. In the dark, COP1 directly targets EBF1/2 for ubiquitination and degradation to stabilize EIN3/EIL1, besides ethylene is produced by the mechanical impedance to promote the accumulation of EIN3/EIL1 by inhibiting the stability and translation of EBF1/2 [78,80,81,82]. The accumulated EIN3/EIL1 and PIFs directly interact with the promoter of *HLS1* (HOOKLESS 1) to additively activate its gene expression and promote apical hook formation (Figure 1) [30,83]. With seedlings successfully protruding through the soil, decreased soil pressure and increased light signal promote the degradation of EIN3/EIL1 and PIFs to accelerate de-etiolation. Besides, red light-activated phyB acts as a scaffold to enhance the interaction of EIN3 and EBF1/2, further accelerating the EIN3 degradation and facilitating photomorphogenesis [84].

Thus, photomorphogenic repressors including COP1, SPAs, CSNs, COP10, DET1, PIFs, and EIN3/EIL1 which are active in the dark to promote seedling skotomorphogenesis, upon light illumination, photo-excited receptors associate with COP1, SPAs, PIFs, and EIN3 to repress their activity or protein accumulation in an effort to promote plant photomorphogenesis through promoting the accumulation of photomorphogenesis-promoting factors such as HY5, HYH, LAF1, HFR1, BBX21, and GATA2. In addition, a large number of BBX proteins have recently been reported to act as intermediates to regulate photomorphogenesis. BBX4, BBX20, BBX21, BBX22, and BBX23 promote photomorphogenesis, whereas BBX18, BBX19, BBX24, BBX25, BBX28, BBX29, BBX30, and BBX31 repress photomorphogenesis [28,85,86,87,88,89,90,91,92,93,94].

## 3. Brassinosteroid Biosynthesis and Signal Transduction

**Figure 3:**
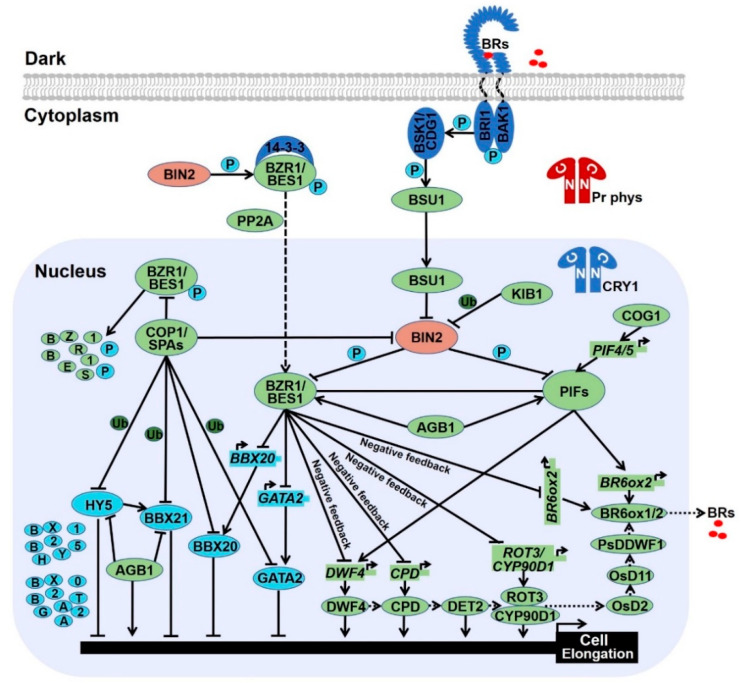
Brassinosteroids regulate skotomorphogenesis in the dark.

As internal signals, BRs are broadly distributed throughout the plant kingdom [4,5]. Over 70 BRs have been identified, among which only brassinolide (BL) and castasterone (CS) are proved to be biologically active. BL, the first purified and structurally determined BR from bee-collected *Brassica napus* pollen grains, is the final and most active product of the BR biosynthetic pathway. CS, an immediate precursor of BL, possesses appropriately 10% of the physiologically activity of BL [95]. Through genetic screening and biochemical analyzing, three major parallel pathways have been identified to synthesize biological BRs, which are the early C-22 oxidation, the early C-6 oxidation, and the late C-6 oxidation pathways [95]. However, plants mainly employ the first part of the C-22 oxidation and the second part of late C-6 oxidation route to synthetize biological BR which only takes 8 steps rather than the 10 steps of the early or the late C-6 oxidation pathways. Key enzymes catalyzing the BR biosynthetic pathway consists of DWARF 4 (DWF4), CONSTITUTIVE PHOTOMORPHOGENESIS AND DWARFISM (CPD), DE-ETIOLATED 2 (DET2), ROTUNDIFOLIA 3 (ROT3) and CYP90D1, OsD2, OsD11, DARK-INDUCED DWF-LIKE PROTEIN 1 (PsDDWF1), and BRASSINOSTEROID-6-OXIDASES 1 and 2 (BR6ox1 and BR6ox2) (Figure 3) [95,96].

Immense studies have illustrated BR signal transduction pathway started by the activation of BRASSINOSTEROID INSENSITIVE 1 (BRI1) and BRI1-ASSOCIATED RECEPTOR KINASE 1 (BAK1) which are the receptor and co-receptor respectively [97,98,99,100,101,102]. Both BRI1 and BAK1 are leucine-rich repeat receptor-like kinases (LRR-RLKs). BRI1 consists of a signal peptide at its N-terminus, followed by an extracellular domain with 25 tandem LRRs, a single-pass transmembrane domain, and a C-terminal cytoplasmic kinase domain. BAK1 is named SOMATIC EMBRYOGENESIS RECEPTOR KINASE3 (SERK3) which is composed of a N-terminal predicted signal peptide, four leucine zippers, five LRRs, a proline-rich region, and a single transmembrane domain, followed by a serine/threonine protein kinase domain [98,99]. Biochemical and structural analysis indicates that the perception of BR causes the conformational change of BRI1 which recruits BAK1. Furthermore, BR acts as the molecule glue to strengthen the interaction and promote the transphosphorylation between the kinase domains of BRI1 and BAK1 [100,101,102], which in turn phosphorylates BR-SIGNALING KINASE 1 (BSK1) at Ser230 and CONSTITUTIVE DIFFERENTIAL GROWTH 1 (CDG1) at Ser234. The phosphorylated CDG1 directly phosphorylates BRI1-SUPPRESSOR 1 (BSU1), resulting in dephosphorylation and repression of the glycogen synthase kinase-3 (GSK3)-like kinase BIN2 [103,104,105]. KINK SUPPRESSED IN BZR1-1D (KIB1), a F-box E3 ubiquitin ligase, interacts with BIN2 to accelerate its ubiquitination and degradation, as well as blocks the association of BIN2 and its substrates [106]. Both BSU1 and KIB1-triggered the inhibition of BIN2 reduces the phosphorylation of BRASSINAZOLE RESISTANT 1 (BZR1) and BRI1-EMS-SUPPRESSOR 1 (BES1) family transcriptional factors [107,108,109]. The dephosphorylated BZR1 and BES1 are biologically active form which control thousands of BR-responsive genes by directly binding to their BR-response element (BRRE, CGTGC/TG) and E-box (CANNTG) motifs, not only promoting cell elongation-related genes such as *IAA19* and *PRE1*, but also confining BR biosynthetic genes such as *CPD* and *DWF4* via a negative feedback regulation [107,109,110,111,112]. Phosphorylated BZR1 and BES1 prefer to interact with 14-3-3 proteins which restrain them in the cytoplasm and accelerate their degradation, while PROTEIN PHOSPHATASE 2A (PP2A) dephosphorylates BZR1 in the nucleus, resulting in the expression of myriad of target genes [107,113,114,115,116].

Taken together, in the absence of BR, BRI1 KINASE INHIBITOR 1 (BKI1) physically interacts with BRI1 and negatively regulates the kinase activity of BRI1 via blocking the binding of BAK1 [117,118]. Meanwhile, the constitutively activated BIN2 phosphorylates BZR1 and BES1 to inhibit their nuclear import ability, protein stability, and DNA-binding activity. In the presence of BR, BRI1 and BAK1 promote the phosphorylation of BSK1 and CDG1. The activated CDG1 phosphorylates BSU1, leading to the dephosphorylation and inhibitory of BIN2 which promote the nuclear accumulation of dephosphorylated BZR1 and BES1. The biologically active form of BZR1 and BES1 stimulate the downstream BR cascade via altering the expression of thousands of target genes to promote skotomorphogenesis (Figure 3).

## 4. Signal Integration of Light and Brassinosteroid

Light and BR oppositely control the development switch from shotomorphogenesis in the dark to photomorphogenesis in the light. Numerous mutants involved in light and BR signals have overlapping phenotype, implicating the link between light and BR are intimate [4,5,119]. Genetic and biochemical evidences demonstrate that light signal share the highly component identity with BR signal to fine-tune plant growth and development.

In the absence of BR, constitutively active BIN2 not only phosphorylates BZR1 and BES1 to restrict their transcriptional activities, but also phosphorylates PIF3 and PIF4 to stimulate their degradation. Phosphorylated BZR1 and BES1 prefer to interact with 14-3-3 proteins which restrain them in the cytoplasm, whereas PP2A-mediated dephosphorylation of BZR1 and BES1 accelerate their nuclear accumulation. In the presence of BR, BR-bound BRI1 recruits BAK1 to initiate their transphosphorylation which activates BSK1 and CDG1. The activated CDG1 phosphorylates BSU1 which in turn dephosphorylates and inactivate BIN2. The inactivated BIN2 releases BZR1 and BES1, as well as PIF3 and PIF4. In addition, AGB1 not only interacts with BES1 to improve the ratio and transcriptional activity of dephosphorylated BES1, but also associates with PIF3 to block its degradation. COG1 directly binds to the promoters of *PIF4* and *PIF5* to upregulate their transcription. PIF4 and PIF5 can directly bind the promoters of *DWF4* and *BR6ox2* to enhance BR biosynthesis. Several critical enzymes have been identified in the BR biosynthesis pathway, including DWF4, CPD, DET2, ROT3, OsD2, OsD11, PsDDWF1, and BR6ox1/2. When the BR reaches certain levels, BZR1 and BES1 arrest the transcriptional levels of *DWF4*, *CPD*, *ROT3*, and *BR6ox1/2* via a negative feedback loop. In the dark, activated COP1-SPAs complex promote skotomorphogenesis by triggering photomorphogenesis-promoting factors such HY5, BBX21, BBX20, and GATA2 for ubiquitination and degradation. Meanwhile, phosphorylated BZR1 and BES1 are degraded in a COP1 dependent manner, resulting in the high ratio of dephosphorylated BZR1 and BES1 which also contribute to skotomorphogenesis.

### 4.1. Photomorphogenic Repressors Elevate Brassinosteroid Response

Pea *DDWF1* and *Pra2* are two light regulated genes involved in BR biosynthesis. Pra2 interacts with DDWF1 to regulate the C-2 hydroxylation activity of DDWF1 which converts 6-deoxotyphasterol (6-deoxoTY) and TY to 6-deoxoCS and CS, promoting BR production [120]. Besides, both *DDWF1* and *Pra2* are dark-induced and light repressed, although how light suppresses the transcriptional level of *DDWF1* and *Pra2* is still unclear. This evidence signifies that light negatively controls BR biosynthesis through arresting *DDWF1* and *Pra2* gene expression in pea. COGWHEEL 1 (COG1), a Dof-type transcription factor, represses light signal in a phyA and phyB dependent manner [121]. Gain-of-function of *cog1-3D* partially suppresses the short hypocotyl of *bri1-5*, while loss-of-function of *cog1-6* exhibits short hypocotyl in the light, indicating COG1 positively regulates BR response but negativity controls photomorphogenesis. Biochemical evidence reflects that COG1 stimulates *PIF4*/5 gene expression via directly binding to the promoter of *PIF4*/*5*, which in turn promote the expression of *DWF4* and *BR6ox2* through interacting with the G-box motif presenting at their promoters [122]. The transcriptional cascade, from COG1 to DWF4 and BR6ox2 which is mediated by PIF4/5, elevates the BR level to allow hypocotyl elongation. Therefore, photomorphogenic repressors COG1 and PIF4/5 function as critical nodes to orchestrate the interconnection between light and BR [122].

AGB1, an Arabidopsis G-protein β subunit, is known to repress photomorphogenesis for the partial de-etiolation phenotype of *agb1* mutant [123,124]. It has been reported that AGB1 represses the expression of *BBX21* and interacts with BBX21 to restrict its transcriptional activity [125]. In addition, AGB1 interacts with HY5 to inhibit its DNA-binding ability, as well as interacts with PIF3 to protect PIF3 from phosphorylation and degradation [126,127]. Both protecting of PIF3 and the inhibitory of HY5 and BBX21 contribute to AGB1-induced skotomorphogenesis. However, the blue light-triggered interplay between CRY1 and AGB1 results in the disassociation of AGB1 from HY5, releasing HY5-mediated genes expression and photomorphogenesis [126]. In the red light, photoactivated phyB competitively binds with AGB1 which removes the protection of PIF3, leading to the phosphorylation and degradation of PIF3 and facilitate photomorphogenesis [127]. It is worth to mention that the role of AGB1 in the light signal may be similar to COP1 which also contains a domain homologous to the β subunit of trimeric G proteins [31]. In addition, AGB1 interacts with BES1 to improve the ratio of dephosphorylated to phosphorylated BES1, as well as synergistically modulates the expression of BES1 targets such as *CPD*, *DWF*, and *SAUR* family genes required for cell elongation, supporting that photomorphogenic repressor AGB1 is a positive regulator of BR response (Figure 3) [128]. Thereby, AGB1 is identified as a junction between light and BR pathways. However, whether AGB1 acts as a bridge to connect BES1 with BBX21 and HY5 to coordinate light and BR cascade remains elusive. BBX32, the last member of BBX family of proteins in Arabidopsis, is a photomorphogenic repressor based on its role in promoting hypocotyl elongation and inhibiting anthocyanin accumulation [129]. A recent study indicates that BBX32 interacts with PIF3 to promote BR-mediated cotyledon closure during transition from dark to light. In addition, BBX32 associates with BZR1 which elevates the expression of *BBX32* to form a positive feedback regulation. Thus, these data suggest that BBX32 acts as a node to integrate light and BR signaling to modulate cotyledon closure during de-etiolation [130].

**Figure 4:**
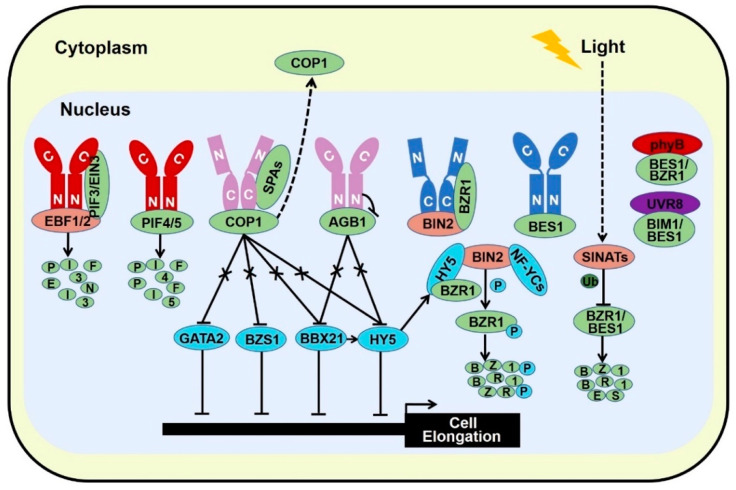
Light and BR share common regulatory components to optimize plant growth and development.

The stability of BES1 and BZR1 is tightly controlled by several E3 ligases. MORE AXILLARY GROWTH LOCUS 2 (MAX2), a subunit of SCF E3 ligase, is critical for strigolactone signaling. BES1 has been reported to interact with MAX2 which promotes BES1 degradation, leading to the inhibitory of shoot branching [131]. PLANT U-BOX 40 (PUB40), a U-box ubiquitin E3 ligase, is responsible for proteasome-mediated degradation of BZR1 in a root-specific manner [132]. SINA OF ARABIDOPSIS THALIANA (SINATs), RING finger E3 ubiquitin ligases, are degraded in the dark, while light promotes the accumulation of SINATs which directly interact with BES1 and BZR1. Biochemical evidences signify that light-stabilized SINATs prefer to bind dephosphorylated BES1 and capture BES1 for ubiquitination and degradation to arrest hypocotyl elongation and BR cascade (Figure 4) [133]. In addition, the phosphorylated BZR1 serves as the substrate of COP1 for degradation in darkness, increasing the ratio of dephosphorylated to phosphorylated BZR1. The relatively accumulated active form of BZR1 results in more active dimers with either dephosphorylated BZR1 or PIF4 to enhance BR response and hypocotyl elongation [134]. Similar with phosphorylated BZR1, COP1 is responsible for the degradation of phosphorylated BES1 in the dark [133]. Therefore, stabilized SINATs mediate the ubiquitination and degradation of dephosphorylated BES1 in the light to promote photomorphogenesis, whereas dark-activated COP1 degrades phosphorylated BZR1 and BES1 to increase the relatively ratio of active form of BZR1 and BES1. Both dark-reduced SINATs protein level and dark-stimulated COP1 activity contribute to the high ratio of active form of BZR1 and BES1 in the nucleus, allowing the BR response and obstructing photomorphogenesis.

Previous data elucidate that BZR1 and BES1 interact with skotomorphogenic-promoted transcription factor PIF4 to synergistically regulate their target genes that are required for cell elongation (Figure 4) [8]. Recently, it is documented that BZR1 and PIF4 promote the gene expression of GROWTH REGULATED FACTOR 7 (*GRF7*) and *GRF8* to repress chlorophyll biosynthesis. Besides, GRF7, BZR1, and PIF4 interact with each other to precisely modulate the greening process via regulating genes involved in chlorophyll biosynthesis. Thus, GRF7-BZR1-PIF4 module orchestrates light and BR signaling pathway to enhance seedling survival during de-etiolation [135]. In addition, BLUE-LIGHT INHIBITOR OF CRYPTOCHROMES 1 (BIC1), which is identified as a repressor of flowering and photomorphogenesis via inhibiting CRY2 phosphorylation, acts as a transcriptional coactivator of BZR1 and PIF4 for the activation of BR-responsive genes to promote hypocotyl elongation. Collectively, BIC1-BZR1-PIF4 complex integrates light and BR response to coordinate plant growth [136]. Moreover, PIF4 along with PIF3 have been reported to serve as substrates of BIN2, which facilitate the phosphorylation and degradation of PIF3 and PIF4 [11,137]; however, COP1-SPA complex interacts with PIF3 and interferes with the BIN2-PIF3 interaction, leading to the accumulation of PIF3 and thus to facilitate skotomorphogenesis [137]. Furthermore, high temperature-promoted nuclear-localized BZR1 binds to the promoter of *PIF4* to promote its gene expression and thermomorphogenesis [138]. Thus, the BIN2-BZR1-PIFs regulatory module integrates hormonal and environmental signals to coordinate plant growth and development.

Photoexcited phytochromes interact with PIF3/4/5 and EIN3 to promote their phosphorylation and/or degradation. Light-activated phytochromes and cryptochromes associate with COP1-SPAs complex to inhibit the E3 ligase activity of COP1 in the nucleus which releases its targets such as HY5, BBX21, BZS1, and GATA2. Red light-excited phyB interacts with AGB1 to disassociate AGB1 from PIF3 and promote PIF3 degradation, while blue light-activated CRY1 binds AGB1 to attenuate its inhibitory effect on HY5 and BBX21 which stimulates photomorphogenesis. Photoactivated CRY1 binds both BZR1 and BIN2, decreasing the transcriptional activity of BZR1. In addition, blue light-dependent interaction of CRY1 and BES1 blocks the DNA binding ability of BES1 and its target gene expression, obstructing brassinosteroid-induced cell elongation. Likewise, red light activated phyB interacts with dephosphorylated BZR1 and BES1, restricting the gene expression of their targets. Moreover, bioactive UVR8 monomer interacts with BIM1/BES1 transcription factors and inhibits their activity to promote UVB-triggered photomorphogenesis. Besides, HY5 interacts with BIN2 and BZR1 to strength the kinase activity of BIN2 on BZR1, diminishing the transcriptional activity of BZR1 in the nucleus. NF-YCs also interact with BIN2 to facilitate its stability by increasing its auto-phosphorylation. Meanwhile, Light-stabilized SINATs promote the ubiquitination and degradation of BZR1 and BES1, reducing BR response and enhancing photomorphogenesis. Red color represents phyA/B, Dark blue color stands for CRY1, and pink color represents both phytochromes and cryptochromes.

### 4.2. Photomorphogenesis-Promoting Factors Repress Brassinosteroid Signal

Recent studies have revealed that blue light receptor CRY1 blocks the BES1 DNA-binding activity and limits its target gene expression via specifically interacting with the physiologically functional dephosphorylated BES1, thereby promoting photomorphogenesis and impeding BR response [13]. Besides, photoactivated phyB interacts with dephosphorylated BES1 to repress its transcriptional activity, allowing plants to balance light and BR signaling [15]. Similar to BES1, blue light-excited CRY1 and red light-excited phyB interact with the DNA-binding domain of BZR1 to reduce its DNA-binding ability and confine the expression of its target genes [14,16]. Moreover, blue light-activated CRY1 interacts with BIN2 to strengthen the physical interaction between BIN2 and BZR1, promoting the phosphorylation of BZR1 and inhibiting its nuclear accumulation, ultimately leading to the impedance of hypocotyl elongation [14]. Therefore, visible light exploits the phyB-BES1/BZR1, CRY1-BES1, and CRY1-BIN2-BZR1 regulatory module to obstruct BR signaling to optimize plant photomorphogenesis. In addition, BR response is also inhibited by UV-B radiation. Similar to phyB and CRY1, UVR8 physically interacts with BES1 INTERACTING MYC-LIKE1 (BIM1) and dephosphorylated BES1. Upon UV-B radiation, monomerized UVR8 and UVR8-BES1 complex are accumulated in the nucleus, leading to the repression of BES1 DNA-binding activity and its target gene expression [12]. Moreover, BES1 directly binds to the promoters of *PFG MYBs* to repress gene expression and constrain flavonol biosynthesis, while broad-band UV-B limits the transcription of *BES1* in a UVR8 independent manner, leading to the accumulation of flavonol and UV-B tolerance [18]. Thereby, UV-B light employs UVR8-BES1/BIM1 interaction and BES1 to orchestrate UV-B signal and BR signal in fine-tuning plant growth.

Moreover, HY5, the famous photomorphogenesis-promoting transcription factor, has been proved to specifically interact with dephosphorylated form of BZR1 and reduce its transcriptional activity, consequently leading to repress the expression of BZR1-controlled genes that are related to cotyledon opening. Ectopic expression of HY5 decreases the abundance of BZR1. Thus, HY5 not only represses the transcriptional activity of BZR1 but also attenuates its protein stability to promote cotyledon opening [139]. However, the physical connection between HY5 and BES1 in the regulation of seedling morphogenesis remains to be dissected. Recently, HY5 has been documented to interact with BIN2 to enhance the kinase activity of BIN2 possibly via improving BIN2 Tyr200 autophosphorylation, ultimately promoting seedling photomorphogenesis (Figure 4) [17].

GATA2 is a transcription factor that regulates genes in response to light via the essential GATA light-response promoter element (LRE) [140]. Knock down of *GATA2* lines show long hypocotyl in the light, while overexpression of *GATA2* confers short hypocotyl and open cotyledon in the dark resembling the light-grown seedlings, indicating that GATA2 is a photomorphogenesis-promoting factor [140]. In the dark, COP1 interacts with GATA2 to facilitate its ubiquitination and degradation, meanwhile, BR confines the transcript level of *GATA2* through the directly binding of BZR1 on the promoter of *GATA2* [140]. Thus, both the low RNA and protein level of GATA2 contribute to etiolation in darkness. Under light treatment, attenuated COP1 activity in the nucleus induces the protein accumulation of GATA2 which in turn negatively regulates its own transcription via the directly binding to the promoter of itself. Such opposite regulatory of transcript and protein levels form a negative feedback loop to maintain a proper protein level of GATA2 for the right photomorphogenesis. Both BZR1 and GATA2 impede *GATA2* gene expression, whether BZR1 interacts with GATA2 to cooperatively regulate the transcription of *GATA2* needs further investigation. Similar with GATA2, knockdown of *BZR1-1D SUPPRESSOR 1 (BZS1)* displays slightly long hypocotyl in the red and blue light, while overexpression of *BZS1* exhibits de-etiolation phenotype in the dark. Such genetic data imply that BZS1 promotes photomorphogenesis [9]. BZS1, also known as BBX20, shows extensive sequence homology to BBX21 [141]. Gain of function of BZS1-D is a dominant suppressor of BR hypersensitive mutant *BZR1-1D*, reflecting BZS1 is a negative regulator of BR signal. Further studies indicate that BZS1 is degraded in the dark via a COP1-dependent manner, whereas BZR1 represses the transcriptional level of *BZS1* which antagonistic controls gene expression involved in light and BR signaling [9]. Thus, light-inactivated COP1 results in BZS1 accumulation to promote photomorphogenesis, whereas BR-activated BZR1 reduces *BZS1* gene expression to alleviate the inhibition of BR cascade. Taken together, GATA2 and BZS1 act as hubs to integrate light and BR signaling (Figure 3 and Figure 4). As the closet homolog of BZS1, BBX21 is critical for photomorphogenesis via enhancing the gene expression and transcriptional activity of HY5 [28,87], however the role of BBX21 in BR signal is vague. Further analysis of the function of BBX21 will add new insights into the crosstalk between light and BR signals.

NUCLEAR FACTOR-Y C PROTEINS (NF-YCs) are subunits of NF-Y heterotrimeric complex that regulate target gene expression by specifically binding to the CCAAT-box-containing promoters [142]. NF-YCs not only inhibit BR biosynthesis by directly targeting the promoter of the BR biosynthesis gene *BR6ox2*, but also interact with BIN2 to promote its Tyr200 autophosphorylation and protein stabilization, resulting in inhibiting the BR signaling pathway during light-controlled hypocotyl growth [19]. Therefore, photomorphogenic-promoting regulators NF-YCs repress BR biosynthesis and signaling to regulate seedling development.

### 4.3. MicroRNAs Integrate Light and Brassinosteroid Signals

MicroRNAs (miRNA) are a group of small, non-coding endogenous RNA molecules (sRNA) that play crucial roles in plant growth and development. Some microRNAs have recently been reported to regulate seedling morphogenesis partially through the regulation of light and brassinosteroid signaling. MicroRNA396a regulates several members of the GRF family at posttranscriptional level. Overexpression of miR396a develops a constitutive photomorphogenic phenotype in the dark, including shorter hypocotyls, opened cotyledons, and highly accumulated Pchlide. Consistently, *grf1/4/7/8* quadruple mutant shows dramatically lower greening rate compared to the wild type, and also exhibits a de-etiolation phenotype in the dark. In addition, GRF7/8, BZR1, and PIF4 interact with each other to form a tripartite module, which positively regulates cell elongation related genes, but negatively controls Pchlide biosynthesis related genes. Collectively, MiR396 represses gene expression of *GRFs* at posttranscriptional level, resulting in promoting photomorphogenesis and repressing brassinosteroid signaling during seedling de-etiolation [135]. Brassinosteroid up-regulates the expression of *MiR395a* which might repress *GUN5* expression, leading to promote the root development [143]. In addition, brassionsteroid decreases the localization of AGO1 at the endoplasmic reticulum (ER), which inhibits the miRNA-mediated translational repression, resulting in increasing the protein levels of miRNA target genes [144]. MicroRNA osa-miR1848 targets *OsCYP51G3* for suppression at posttranscriptional level. *OsCYP51G3* encodes an obtusifoliol 14α-demethylase which positively regulates phytosterol and brassinosteroid biosynthesis. Increased osa-miR1848 and decreased *OsCYP51G3* expression cause a BR deficiency phenotype, including dwarf plants, semi-sterile pollen grains, and erect leaves. Thus, osa-miR1848 negatively regulates brassinosteroid biosynthesis to control plant architecture, which has potential to be utilized in rice breeding by adjusting leaf angle, plant height, and seeds quality [145].

Overall, in the absence of BRs, the constitutively activated BIN2 not only phosphorylates and inactivates BZR1/2, but also phosphorylates PIF3/4 which result in the degradation of PIF3/4, consequently to promote photomorphogenesis [11,137]. In the presence of BR, inactivated BIN2 not only relieves BZR1/2, but also releases PIF3/4. The stabilized and activated PIF3/4 and BZR1/2 act in concert to promote downstream gene expression to stimulate skotomorphogenesis/etiolation. In the dark, activated COP1 targets phosphorylated BZR1 and BES1 for ubiquitination and degradation, leading to the high ratio of active form of BZR1 and BES1 [133,134]. Besides, COP1-SPA complex interacts with PIF3 and blocks the BIN2-PIF3 interaction, resulting in the accumulation of PIF3 [45,68]. Moreover, brassinosteroids receptors perceive signal at the cell surface and deliver it to BZR family transcription factors and PIF transcription factors in the nucleus respectively. Both the high ratio of dephosphorylated BZR1/2 and high protein level of PIFs contribute to facilitate skotomorphogenesis (Figure 3). However, in the light, photoreceptors employ light-promoted transcription factors such as HY5, BBX21, BZS1, and GATA2 to stimulate photomorphogenesis by directly regulating hypocotyl-related gene expression or enhancing the kinase activity of BIN2 or any other unknown mechanisms. Besides, photoreceptors also directly interact with COP1-SPAs complex, AGB1, PIFs, and BZR1 family transcription factors to repress their activity and/or stability to facilitate photomorphogenesis (Figure 4).

## 5. Conclusions and Future Perspectives

Plants have evolved precisely regulatory network to respond external stimuli and internal phytohormonal cues. Both light and brassinosteroids are crucial external and internal signals to determine developmental program. Numerous components have been identified as hubs to integrate light and BR signals. Many reports elucidate that BR signal has been obstructed by photomorphogenesis-promoting factors, including photoreceptors phyB, CRY1, and UVR8, positive transcriptional factors HY5, BBX21, BZS1, GATA2, and NF-YCs, and RING finger E3 ubiquitin ligase SINATs. For example, biological active forms of photoreceptors interact with BZR1 and BES1 to restrict their DNA binding ability, hampering BR response and promoting light signal. Besides, activated CRY1 also binds negative regulator of BR signaling such as BIN2 to enhance its kinase activity on BZR1 and BES1, reducing the active form and nuclear accumulation of BZR1 and BES1, thus impeding BR signaling and elevating photomorphogenesis. However, whether phytochromes modulate the activity of BIN2 is still unclear. HY5 interacts with dephosphorylated BZR1 to restrain the transcriptional activity of BZR1, moreover, the interaction of HY5 and BIN2 strength the kinase activity of BIN2 on BZR1 and repress the accumulation of dephosphorylated BZR1, ultimately arresting BR cascades and facilitating de-etiolation. Besides, NY-YCs repress the expression of *BR6ox2*, as well as associate with BIN2 to facilitate its autophosphorylation and stability, thus inhibiting BR biosynthesis and signaling pathways to promote seedling photomorphogeneis. Whether other transcriptional regulators such as BBX21, BZS1, and GATA2 associate with BIN2 or BES1/BZR1 to orchestrate light and BR signals awaits further study. In addition, light promotes the accumulation of SINATs in the nucleus which accelerate the degradation of dephosphorylated BES1 and BZR1, attenuating BR-triggered etiolation and allowing photomorphogenesis. However, whether light-mediated stabilization of SINATs dependent on phytochromes or cryptochromes or other light regulators remains to be dissected.

BR signals have also been proved to be elevated by photomorphogenic repressors, including COP1, PIFs, AGB1, and COG1. In the dark, phosphorylated BZR1 and BES1 undergoes COP1-dependent degradation, increasing the ratio of dephosphorylated to phosphorylated BZR1/BES1 which enhances BR response and etiolation. Both BZR1/BES1 and PIF3/4 are substrate of BIN2; however, BR-inhibited BIN2 releases BZR1/BES1 and PIF3/4 to stimulate skotomophogenesis. In addition, COP1-SPAs complex blocks the interaction of BIN2 and PIF3 which contributes to PIF3 accumulation. Moreover, PIF4 interacts with BZR1 and BES1 to cooperatively regulate gene expression required for etiolation. Investigating whether COP1-SPAs complex affects the association of BIN2 and BZR1/BES1 is of great interest. AGB1 interacts with BES1 to enhance the transcriptional activity of BES1, as well as improve the ratio of active form of BES1, thus reinforcing BR cascades. COG1 directly binds to the promoter of *PIF4* and *PIF5* to activate their expression. In turn, PIF4 and PIF5 up-regulate the transcriptional level of *DWF4* and *BR6ox2*, resulting in elevating BR production and promoting BR-induced etiolation.

In addition, light signal has also been modulated by BR components. For example, BZR1 represses the transcriptional level of photomorphogenesis-promoting genes such as *GATA2* and *BZS1*. BIN2 phosphorylates PIF3 and PIF4 to promote their degradation which reinforce photomorphogenesis. Likewise, BZR1/BES1 interact with PIF4 to coordinately control gene expression required for etiolation. However, what is the relationship among HY5, BZS1, and GATA2 in orchestrating light and brassinosteroid signals? Whether other BR components such as BRI1, BAK1, CDG1, and BSU1 join in the light signal to adjust developmental procedure remains vague. Dissecting these processes would add new insights into the integration of light and brassinosteroid signals in plant growth and development.

Both light- and brassinosteroid-mediated seedling development are essential regulatory processes that also modulate the crop establishment. Tomato SIBBX20 promotes carotenoid biosynthesis by binding to the promoter of *PHYTOENE SYNTHASE 1(PSY)* which is a carotenoid biosynthesis gene [146]. Apple MdBBX22 interacts with MdHY5 to facilitate the binding ability of MdHY5 to the promoter regions of *MdMYB10* and *MdCHS*, which in turn promoting their expression [147]. Besides, MdHY5 promotes the expression of *MdBBX33*, which modulates *MdMYBA* expression to promote anthocyanin accumulation in the light [148]. Pear PpHY5 directly binds to the G-box cis element within the promoters of *PpMYB10* and *PpBBX18* to induce anthocyanin accumulation [149]. However, PpBBX21 represses anthocyanin biosynthesis by associating with PpHY5 and PpBBX18 to interfere with the physiological active heterodimers of PpHY5-PpBBX18 [150]. Overexpression of *AtBBX32* in soybean elevates the grain yield likely due to increases in key yield components such as pod number, seed number, and individual seed weight per plant [151]. Thus, the integrators of light and brassinosteroid signaling are also key regulators of crop establishment. Additional integrators of light and brassinosteroid signaling are urgently need to be found. Furthermore, it is the time for plant biologists to exert the knowledge of molecular basis of the crosstalk between light and brassinosteroid to improve crop breeding and crop yield.
